# Systemic structural gender discrimination and inequality in the health workforce: theoretical lenses for gender analysis, multi-country evidence and implications for implementation and HRH policy

**DOI:** 10.1186/s12960-023-00813-9

**Published:** 2023-05-04

**Authors:** Constance Newman, Alice Nayebare, Ndeye Mingue Ndiate Ndiaye Gacko, Patrick Okello, Abdou Gueye, Sujata Bijou, Selly Ba, Sokhna Gaye, N’deye Coumba, Babacar Gueye, Yankouba Dial, Maimouna N’doye

**Affiliations:** 1 Department of Maternal and Child Health, UNC Gillings School of Global Public Health, University of North Carolina, 135 Dauer Drive, Chapel Hill, NC United States of America; 2 Cordaid Uganda, Plot 12B Farady Road Bugolobi, Nakawa Division, Kampala, Uganda; 3 Gacko Consulting, Formerly Ministry of Health and Social Action, Fann Résidence, Rue Aimé Césaire, Dakar, Senegal; 4 Ministry of Health, Plot 6, Lourdel Road, Wandegeya, P.O Box 7272, Kampala, Uganda; 5 Formerly IntraHealth International, Cité Keur Gorgui, Immeuble El Hadji Bara Fall Lot R73, Dakar, Senegal; 6 Intrahealth International, 6340 Quadrangle Drive, Suite 200, Chapel Hill, NC 27510 United States of America; 7 Independent Consultant, Dakar, Senegal; 8 Ministry of Health and Social Action, Fann Résidence, Rue Aimé Césaire, Dakar, Senegal

**Keywords:** Systemic structural gender discrimination, Gender inequality, Health labor market, Gender transformative policy, Nondiscrimination and substantive equality

## Abstract

This commentary brings together theory, evidence and lessons from 15 years of gender and HRH analyses conducted in health systems in six WHO regions to address selected data-related aspects of WHO’s 2016 Global HRH Strategy and 2022 Working for Health Action Plan. It considers useful theoretical lenses, multi-country evidence and implications for implementation and HRH policy. Systemic, structural gender discrimination and inequality encompass widespread but often masked or invisible patterns of gendered practices, interactions, relations and the social, economic or cultural background conditions that are entrenched in the processes and structures of health systems (such as health education and employment institutions) that can create or perpetuate disadvantage for some members of a marginalized group relative to other groups in society or organizations. Context-specific sex- and age-disaggregated and gender-descriptive data on HRH systems’ dysfunctions are needed to enable HRH policy planners and managers to anticipate bottlenecks to health workforce entry, flows and exit or retention. Multi-method approaches using ethnographic techniques reveal rich contextual detail. Accountability requires that gender and HRH analyses measure SDGs 3, 4, 5 and 8 targets and indicators. To achieve gender equality in paid work, women also need to achieve equality in unpaid work, underscoring the importance of SDG target 5.4. HRH policies based on principles of substantive equality and nondiscrimination are effective in countering gender discrimination and inequality. HRH leaders and managers can make the use of gender and HRH evidence a priority in developing transformational policy that changes the actual conditions and terms of health workers’ lives and work for the better. Knowledge translation and intersectoral coalition-building are also critical to effectiveness and accountability. These will contribute to social progress, equity and the realization of human rights, and expand the health care workforce. Global HRH strategy objectives and UHC and SDG goals will more likely be realized.

**Supplementary Information:**

The online version contains supplementary material available at 10.1186/s12960-023-00813-9.

## Introduction

This commentary contributes to understanding important gender-related aspects of the World Health Organization’s (WHO) 2016 *Global Strategy on Human Resources for Health* (HRH) and its implementation guidance, such as the 2022 *Working for Health* (W4H) *Action Plan* [1, 2]. It considers theoretical perspectives and evidence from 15 years of gender and HRH analyses conducted in countries in the six WHO’s regions [3] to strengthen data on human resources, align investment in human resources for health to address shortages with particular focus on the *gender dynamics* in health labor markets [1]. Robust data and evidence on gender and HRH have been generally lacking [4]. The 2022 Working for Health (W4H) Action Plan recognizes the potential of the health workforce to accelerate transformational social progress, equity and the realization of human rights. It highlights the need to generate data and use evidence to inform and drive decision-making [2]. It also makes gender equality central to the Working for Health agenda [2, p. 13].

To achieve transformational social progress, equity and the realization of human rights, our understanding of gender equality and inequality in HRH should be informed by evidence, as must HRH policy. Both the theoretical/conceptual and evidence bases of gender and HRH should be strengthened to demonstrate the ways in which *systemic structural gender discrimination and inequality* (SSGDI) are dysfunctions that undermine gender equality, social progress and health/HRH systems’ objectives. In this commentary, we consider theoretical lenses for gender and HRH analysis, multi-country evidence of SSGDI and implications for implementation and policy. We address the following questions:

1. What are systemic structural gender discrimination and inequality (SSGDI) in the health workforce and why are they out of the awareness of most human resources (HR) planners, managers and service providers?
2. Why should it be a priority to collect data to make SSGDI visible in workforce systems?
3. What are the implications for implementation and HRH policy?
4. What are practical steps to make gender analysis results accessible to and actionable by HRH/M policy planners and managers?

## What are systemic structural gender discrimination and inequality (SSGDI) in the health workforce?

### Theoretical lenses for gender and HRH analysis

Theories and concepts from the sociology of gender, work and organizations describe some of the dynamics of gender discrimination and inequality in the health workforce. These can be applied as lenses through which to understand evidence generated by gender and HRH analyses, allowing policy planners and managers to connect the dots between gender dynamics and negative/dysfunctional HRH effects and consequences. Below, we highlight aspects of theory or concepts that contribute to understanding elements of *systemic structural gender discrimination and inequality* and provide illustrative examples in HRH systems. See Box 1 defining *systemic structural gender discrimination and inequality in health education and employment systems.*

*Gender* has been defined differently over disciplines and contexts, with some seemingly irreducible differences and some common elements [5, 6]. One commonality is that gender is a *socially constructed* phenomenon [7]. For this paper, definitional elements should explain the gendered processes and mechanisms operating in the health workforce, for example, its highly gendered composition and occupational structures [4]. The WHO 2022 *Health Labour Market Analysis Guidebook* refers to gender as “*Socially constructed identities, attributes and roles for women and men and society’s social and cultural meaning resulting in hierarchical relationships between women and men and in the distribution of power and rights. This social positioning of women and men is affected by political, economic, cultural, social, religious, ideological and environmental factors and can be changed by culture, society and community* [8, p. 167]. This definition is compatible with the sociological analysis of sociocultural, historical, structural and relational drivers in *systemic, structural gender discrimination and inequality* (SSGDI) in health systems. See Box 1.

Sociological-relational theories give a central place to the small-scale behaviors and social interactions and large-scale patterned relations between women and men (and among women and among men) that constitute gender as a social structure, and which are shaped by, address, and modify this social structure [9]. Gender relational theory encompasses economic, power, affective and symbolic relations, operating simultaneously at intrapersonal, interpersonal, institutional and society-wide levels [9]. Social structures exist in the larger societal *gender order* (e.g., families) and in organizational *gender regimes* (e.g., hospitals). Gender is brought into social relations through interactions [10] that are infused by systems of beliefs and ideology. For example, patterned relations between men and women in the health sector generally reflect the *ideology of domesticity* [11, 12], where nurses do “emotional work” with clients and or are expected to serve physicians [13].

It has been observed that *«gender is one of the three or four identities (including class, race, religion) that are central to the process by which people render themselves comprehensible to themselves and others in terms that are socially valid within their society*…As a result, both men and women have deep cognitive interests in maintaining a clear and reasonably stable framework of gender beliefs that define “who” men and women “are” by differentiating them» [14, 15]. An HRH example of deep cognitive interests in maintaining the gender status quo was demonstrated in a special gender analysis of occupational segregation conducted in HIV/AIDS care and support in Lesotho, where men were publicly ridiculed for engaging in the female-identified tasks involved in childrearing [12]. Even though «gender» may change [8] over time, the strength of societal/institutional interests in preserving frameworks of gender belief and social identity should not be underestimated.

Gender systems not only constitute people as significantly different in essence, but also as of greater or lesser value. Social relations of inequality are then organized in hierarchies on the basis of greater or lesser value [14, 16]. The apparent human tendency to differentiate by supposed essential traits and to stratify by superior–inferior positioning in structural inequality is exemplified by the gender segregation of *occupations*, one of the most profound, widespread and enduring dimensions of labor market inequality, compared with segregation by race or class [17]. An example of a large-scale pattern of hierarchical gender inequality is evident in a sector where care is *delivered by women but led by men* [4]. The deference expected from nurses, midwives and other paramedical cadres in relation to physicians is transmitted through beliefs about differential value and power during the socialization of students in the *gender regimes* of health education institutions [13]. Violence, coercion and harassment also underpin vertical segregation and gender status maintenance in the gender regimes of health education and employment institutions. For example, a gender and HRH analysis of sex-based harassment in Uganda found that intimidation, demands for sex, coercion and gender subordination were common management/supervisory behaviors in staff recruitment, promotion and performance evaluations [18].

Other structural inequalities include the *glass ceiling* and the *glass escalator*, aspects of *occupational segregation *in the health sector. Glass ceiling practices consist of invisible barriers to reaching top leadership and management positions, that include, for example, the initial placement of (usually) women in relatively low-level, dead-end jobs; not offering job assignments that lead to advancement; not promoting women or greater scrutiny on women’s performance relative to men’s before being promoted; or a lack of access to informal networks and opportunities for mentoring [19]. A gender and HRH analysis in the public health sector of Uganda demonstrated how a *glass ceiling* was generated (perhaps unintentionally but exclusionary in effect) during recruitment by a criterion for a leadership and management position required the job candidate to have a medical degree to be selected—a condition to which the majority-female nurse workforce in Uganda was unable to comply [50]. The *glass escalator* refers to a workforce effect whereby men bring their privileged status from the wider society/culture to their entry into predominantly female occupations. Male nurses are often accepted and well-integrated in a female-dominated profession such as nursing and given fair if not preferential treatment in hiring and promotion decisions, despite their being in a minority [20].

Both the *glass escalator* and *glass ceiling* are linked to notions of the greater status worthiness of men (i.e., *male primacy*) [16], for example, the presumption of greater male competence in leadership. These dynamics were made clearly visible in a 2019 global gender and HRH analysis of occupational segregation in nurse leadership [13]. This analysis also found that pregnancy and family responsibilities, in the absence of childcare, was a key contributor to women’s time poverty, a material barrier to seeking more education and credentials which are part of career progression. Pervasive, intense *gender-relational* stereotyping and “*prove it again*” dynamics [21] for women underpinned a *glass escalator* in recruitment and promotion of male nurses, which served to exclude female nurses from senior leadership roles in service delivery, research, and health policy.

The foregoing examples demonstrate the processes of gender differentiation, stratification, coercion/violence and exclusion that are aspects of *systemic structural gender discrimination and inequality* in HRH systems.

Box 1. Systemic structural gender discrimination and inequality in health education and employment systems The term *systemic, structural gender discrimination and inequality* (SSGDI) covers widespread, but often masked patterns of gendered behavior, policies or practices, relations and the social, economic or cultural background conditions that are entrenched in the processes and structures of institutions (such as health education and employment institutions) and which can create or perpetuate disadvantage for some members of a marginalized group relative to other groups in society or organizations [22–24]. SSGDI.

- Is created through past/historic discrimination in gendered social orders and in institutional gender regimes
- Includes direct and indirect discrimination
- Is the most persistent obstacle to the achievement of substantive gender equality
- Underscores the ineffectiveness of gender-blind policies that do not recognize gender and that women and other marginalized groups may continue to deal with the effects of past or historic discrimination in the present, while others may continue in the present to benefit from historic social advantage [22–24].

## Why are systemic structural gender discrimination and inequality (SSGDI) out of the awareness of most human resources (HR) planners, managers and service providers?

The dynamics of SSGDI are usually *masked or invisible* unless concerted efforts are made to make them visible. They are masked because they are expressions of everyday behaviors and norms which are central to the smooth operation of small-scale interpersonal interactions or large-scale institutional patterns in gender systems. Often, masculine-typed behaviors are taken as the norm—either as universal or normalized. For example, an analysis of sexual harassment in Uganda’s public health sector [18] showed that unwanted sexual attention towards female health workers and body-shaming were parts of day-to-day staff interactions in workplaces with no policy to regulate such behaviors. *Gender-blind* institutional policies also ignore and mask sexually harassing behaviourial norms and may contribute to maintaining class and, possibly, race and ethnic, stratification, which may also be masked in organizations [25].

### Gender blindness

The term «gender blindness» («Gender neutrality» in human rights and sociological literature) describes why/how SSDGI operate out of the awareness of most HR planners, managers and service providers. It refers to «*behaviors or policies that ignore gender norms, roles and relations and very often reinforce gender-based discrimination. By ignoring differences in opportunities and resource allocation for women and men, such policies are often assumed to be “fair” as they claim to treat everyone the same.*» [7]. For example, a wage policy can try to be fair by being “blind” with respect to gender and yet implicitly reward a assumed gendered “breadwinner” in the household.

### Health organizations are gendered and jobs embodied

It is not unusual to be *blind* to the gendered nature of *organizations* and *jobs*. Common understandings of the «job» are often based on an implicit assumption that there is an abstract, disembodied *universal worker* who can dedicate 100% time to the job unencumbered by child-bearing/rearing, a worker (usually a man) who can depend on someone else to play the gendered role of a «wife» who takes care of domestic needs, children and other family responsibilities. The conception of an abstract, disembodied universal worker in a disembodied job nevertheless operates through a gender-based division of labor and the separation between the public and the private spheres which are evident in the masked but widespread phenomenon of occupational segregation [25]. Implicit in the abstract *universal worker* are actual preferences for men’s expectations of paid work in the public sphere and looser relationships to procreation and labor in the private sphere (not to mention so-called masculine leadership traits and higher representations of men in senior leadership) [25]. Implicit assumptions of an abstract job underpin workplace norms of full-time labor market availability to which men can more easily comply, which ultimately marginalize women in the gendered organization [25].

*Gender-blind language* such as «health workers» also masks the reality of a gendered relationship to public and private spheres. For example, a time-use survey comparing the public- and private-sphere workloads of female and male full-time health workers in Mali found that female health workers had more domestic responsibilities than male health workers and that these took more time (e.g., cooking, washing dishes feeding and caring for children and spouses and family, breastfeeding, supervision of children's homework, housekeeping, etc. [45, 46]), essentially another full-time job.

### Social embodiment

It should be noted that health jobs are occupied by *embodied* workers with the potential to procreate, among other things. At a minimum:

*«…All serious gender theory concerns bodies. Gender is a structure of a specific kind, built from the relationships that concern the reproductive distinctions between human bodies. Gender practice is a reflexive process of social embodiment…how a society handles sexuality, reproduction, child growth, motherhood, fatherhood, and all that is socially connected with these processes.»* [9, p. 1677]

That health labor market jobs involve *social embodiment* is typically ignored in gender-blind HR policies and practice. For example, a gender and HRH analysis in Senegal’s public health sector (which requires continuous operation) found that night duty was delegated to nurses and midwives the majority of whom were married women of reproductive age with children, separated by deployment policy from spouses in rural areas with no institutional or familial childcare support. To manage the conflicts created by tending to private-sphere responsibilities at night, these *health workers* were often obliged to bring their infants along with them into the public sphere where night guard duty took place. Failure to factor *embodiment* and public and private sphere workloads into HRH policies, job design, schedules and workloads is at the heart of the widespread problem of «work–life conflict» and attrition.

Some managers view human reproduction as an intrusion into the workplace and respond not by proactive planning but by excluding (not hiring or promoting) women of reproductive age from good jobs in the labor market. Research demonstrates that family caregiving excludes women from health education and training [26, 45, 46, 48] and that pregnancy has been used to legitimize firing or demotion in the name of organizational efficiency [27; Also, Additional file 1].

### Combined effects and consequences: Social closure

Finally, the concepts of *social closure* and *social closure discrimination* [28] are useful to understand the combined effects and consequences of the processes in small- and large-scale, yet masked interactions and patterns that affect health labor market participation, and which include relational stereotyping, sexual harassment, pregnancy and reproductive role discrimination (sometimes referred to as the «maternal wall» [19]), the glass ceiling and escalator and the gender segregation of occupations [28]. *Social closure* involves the drawing of boundaries between groups in “*a process of subordination in which one group secures its advantages by closing off the opportunities of another group that it defines as inferior and ineligible*”, and by maximizing rewards by restricting access to resources and opportunities to a limited circle of eligibles» [28, 29] in masked or invisible *inequality generating processes* [30, 31]. They are involved in closing off occupational and professional opportunities in which female health workers are relegated to “occupational ghettoes” in low-status, lower-paying jobs, with fewer prospects for career advancement in labor markets [16].

### Evidence of systemic gender structural discrimination and inequality

Inequality generating processes of gender differentiation, stratification, coercion/violence, exclusion and social closure are at work in *systemic structural gender discrimination and inequality* (SSGDI) in the health workforce. Evidence in Table 1 shows how widespread patterns of gendered interactions/relations act as HRH dysfunctions with negative effects on health workers’ career and economic prospects, as well as on the achievement of health systems’ objectives.

**Table 1:** Evidence of systemic structural gender discrimination and inequality and negative HRH effects and consequences from country and global gender analyses

Country/focus	Systemic structural gender discrimination	Negative HRH effects and consequences
Senegal (2021): Deployment, family separation, work–life conflict, absenteeism and sexual violence [50]; Supporting Evidence File	Constellation of discrimination: vertical and horizontal and task segregation, reproductive role/status discrimination; multi-source violence and sex-based harassment; victim-blaming and silencing; men share selected family/domestic tasks, but are women responsible, valued for *domesticity*; non-recognition/devaluation of women’s paid health work and economic contribution to household; full- and (unpaid) overtime work; night duty; no childcare; marital/familial conflict; non-respect of maternity leave and breastfeeding; non-hiring and promotion of pregnant workers; male preference and delegitimization of women’s leadership competence/men’s refusal to be supervised or treated by women; *category bias* in access to scholarships, housing, safe and secure work, strategic leadership positions; *maternal wall* and *glass ceiling* practices around recruitment and promotion. Non-application of existing labor protections. “Ideal worker” and gender-blind HRM	Conflict and stress and absenteeism; lack of safety; unequal opportunity for jobs and career; under-representation of women in strategic leadership. Social closure and resignation/exit. Female health workers absorbed back into unpaid care/domestic labor. Workforce shortages, disruption in continuity of services
Global (2019) Nursing Now Gender Analysis of occupational segregation in nursing leadership [13]	Intense gender stereotyping (Gender essentialism and male primacy*)* and devaluation of nursing that maintains doctor/nurse hierarchy; constellation of discriminations, etc.; inflexible hours and the *second shift* after work, time poverty lack of childcare; *glass escalator* for male nurses in promotion, *glass ceiling* for female nurses; under-resourced jobs	Strain, burnout; nurses defer leadership responsibility until children are grown; social closure, workforce exit
Mali (2018): General gender discrimination and inequality analysis in education and employment systems [43]	Pregnancy and family responsibilities-based exclusion/discrimination in both education and employment systems; sexual coercion and *sex-based grades*; lack of safety; women responsible for almost all household labor as well as 100% employment in the labor market, yet underestimation of women’s work and financial contribution to household. No childcare	Conflict, strain, burnout from competing demands; drop out/reduced health worker pipeline
Uganda (2017): Sexual Harassment Formative Assessment [18]	Sex-based harassment (e.g., *gender harassment, unwanted sexual attention* and *sexual coercion*): unwanted sexual attention and body-shaming by colleagues; sexual coercion by managers/supervisors including bullying, gaslighting, victim-blaming and silencing dynamics; punitive transfers; mutually reinforcing intersections of sex-based harassment and vertical occupational segregation as related obstacles experienced by women seeking professional advancement; *sextortion/exploitation* of health workers and patients	Corruption of HRM/supervision; hostile work climate; anxiety, depression and fear; lack of safety; absenteeism; unequal opportunity for advancement; punitive transfers, turnover or exit; damage to organizational reputation; disruptions in continuity and use of services
Ethiopia (2017): Occupational segregation in supply chain management [44]	Gender stereotyping of the supply-chain management (SCM) job, male-targeted recruitment; Pregnancy and family-based exclusions; vertical and horizontal segregation, etc.; and the *second shift* preclude taking on leadership roles; fear of community violence. Non-application of existing labor protections	Women not recruited for SCM field work or management, remain in office jobs. Supply chain inefficiencies
Kenya (2016): General gender discrimination and inequality analysis in medical education systems [45, 46]	Pregnancy and family responsibilities discrimination; women responsible for almost all household labor as well as employment in the labor market; time poverty; familial conflict; *sex-based grades.* Vertical, horizontal and task segregation for students and faculty	Lack of safety; fear; lack of opportunity to advance; social closure and drop out; reduced health worker pipeline
Zambia (2013) and Uganda (2012) General gender discrimination and inequality analyses in small private/large public employment systems [47, 48]	Vertical and horizontal segregation; *maternal wall* and *glass ceiling practices*; “*ideal worker*” norm; denigration of women’s/leadership competency; sexual coercion by supervisors; intrusion of cultural patterns into HR processes; stigmatization of affirmative action. Non-application of existing labor protections	Unequal opportunity for employment; under-representation of women in leadership
Lesotho (2009): Occupational segregation in community-based HIV/AIDs care [12]	Gender essentialist and male primacy stereotyping keep women in caregiving jobs and men out of them; fear of men’s sexual predation against female PLWHA; men refuse volunteer/unpaid work	Unsuccessful recruitment of men; workforce shortages
Rwanda (2008): Workplace violence [49]	*Constellation* of discriminations and violence: verbal abuse, physical assault, bullying, sexual harassment; denigration of women’s/leadership competency; “hostile animus” towards female health workers; pregnancy discrimination; *maternal wall* and *glass ceiling* practices	Strain; lack of safety; “indecent” work

### Sources of data

The evidence of SSGDI in Table 1 comes from a program of multi-country gender and HRH policy research undertaken by IntraHealth International in nine (9) USAID-funded country projects in sub-Saharan Africa between 2009 and 2021, as well as from a 2019 Johnson and Johnson foundation-funded gender analysis of occupational segregation conducted in collaboration with the global Nursing Now campaign. The global nursing survey provided responses from nurses practicing in 117 countries [13] that are included in WHO’s regional groupings [3]: African Region (AFR); Region of the Americas (AMR); South-East Asian Region (SEAR); European Region (EUR); Eastern Mediterranean Region (EMR); and Western Pacific Region (WPR). Five of the countries in Table 1 appear on the WHO *Health Workforce Support and Safeguards List*, countries which face the most pressing health workforce challenges related to Universal Health Coverage [32].

The methodological approach taken in the multi-method analyses summarized in Table 1 (termed *Gender Discrimination and Inequality Analysis* or *GDIA*) anticipated and addressed the need for evidence especially from low- and middle-income countries (LMICs) on the gender dimensions of the health workforce [4]. The GDIAs employed focus groups, key informant interviews, (on- or offline) surveys, analysis of personnel data and document reviews, yielding sex- and age-disaggregated and gender-descriptive data. Some GDIAs (Mali and Senegal) featured time-use surveys to compare the time spent by male and female health workers in paid health, unpaid care and domestic labor.

Qualitative techniques from ethnography (e.g., detailed narratives in «*thick description*» [33] were tested and documented to evoke health workers’ beliefs and perceptions and to generate enough context-specific evidence to inform relevant HRH policy development [8] and implementation, and to assess the *cross-cultural portability* of some aspect of gender-related theory developed in the United States, the United Kingdom and parts of Europe where gender and workforce studies found early traction. Additional file 1, *Systemic structural discrimination and inequality in Senegal’s public health sector*, discusses the meaning of GDIA data in terms of sociological-gender concepts and substantiates their cross-cultural portability.

### Highlights of Table 1

*Systemic, structural gender discrimination and inequality* in or between educational and health employment systems appear across national/sociocultural and political contexts. The GDIAs demonstrate organizational processes of gender differentiation, stratification/subordination coercion/violence, exclusion that close off career paths and relegate female health workers to lower-level sex-typed jobs. Occupational and/or task segregation existed in a *constellation* of other forms of gender discrimination and inequality. Table 1 suggests the near-ubiquity of reproductive status and role discrimination, gender stereotyping and work–life conflict, where female health workers juggled the unequally shared demands of unpaid family care and domestic work and full-time health job responsibilities. Work–life conflict destabilized marital, familial and professional relations. Occupational and career barriers closed off career advancement (i.e*.*, *social closure*). Among other factors, undesirable or unsafe tasks often performed during unpaid night work together suggested a *category bias* against nurses and midwives [34] in Senegal’s public health sector. The dynamics of *male primacy* in the glass ceiling and glass escalator, the devaluation or denigration of women and their competence, and the contesting of women’s authority or presence in workplaces, were underpinned by beliefs about female incompetence (Rwanda) or an *ideology of domesticity* (Lesotho, Senegal), made visible by the GDIAs.

Sex-based harassment (including gender harassment, sexual coercion, quid pro quo and exploitation) or fear of sexual violence and retaliation occurred in all nine [9] country health systems studied. For example, the consequences of “*sex for grades*” in preservice education systems were lack of safety, student drop-out and reduced health worker pipelines (Mali, Kenya). *Quid pro quo* and sexual coercion (Uganda) operated as “*sex for employment rewards*” which created hostile work climates and conflicts between supervisor and staff or between colleagues, barriers to career advancement and turnover and sometimes resulted in workforce exit and discontinuity of health services. Sexually harassing behaviors were typically normalized, silenced by victim-blaming and stigma, not reported and thus obscured in daily work interactions, though also considered an “open secret” (Uganda, Kenya, Senegal).

The negative HRH effects and consequences summarized in Table 1 occurred in employment and education systems where *gender-blind* HRM policy environments allowed gender bias, discrimination and inequality and social closure to operate freely. All these factors were obstacles to health workforce development, retention and expansion—and to the economic security of female health workers.

## Why should it be a priority to collect data to make SSGDI visible in workforce systems?

As seen in Table 1, evidence from gender and HRH analyses can demonstrate how SSGDI work and their undesirable HRH effects and consequences. Once these are understood, the HRH/M policy planner or manager can anticipate certain bottlenecks to workforce entry, internal flows, and exit. Better evidence can make HRH/M policy action more proactive and effective. For example, if jobs are understood to be occupied by *embodied* workers, then lifecycle events would more likely be planned for and staff shortages (temporary or permanent) mitigated through, for example, a pregnancy cover policy. Health service discontinuities would likely be mitigated. If enabling conditions, such as access to childcare, were routinely offered as HRM infrastructure, perhaps female health workers would less easily become targets of marginalization. Parental leave might be an incentive to sharing the burden of unpaid care and domestic work, which would promote equity and social progress, since *to achieve gender equality in paid work, women need to achieve equality in unpaid work* [35]. Any of these evidence-informed policies would likely alleviate the tension, conflicts, burnout and the ad hoc crisis management that often attends family-related absences from work. Policies to prevent sex-based harassment of employees and clients would make professional environments less hostile and exclusionary, improve teamwork, reduce health worker turnover or resignations, improve the poor reputation of health facility and likely increase the utilization of health services [18].

## What are the implications for implementation and HRH policy?

### Implementation

1. To draw strong linkages between SDG5 targets and non-health Sustainable Development Goals (SDGs): gender and HRH analyses must be conceptually stronger, produce better data, and enable policy planners to connect the dots between evidence of an HRH problem and corrective policy action. All SDG 5 targets (Box 2) are relevant to HRH. Stronger data related to SDG 5.4 are needed to supplement data related to SDG 5.1 and 5.2, which together will strengthen our understanding of the interplay of discrimination and inequalities and how to counter *systemic, structural gender discrimination and inequality*.
  Gender and health workforce analyses provide opportunities to strengthen intersectoral linkages and impact non-health SDGs, for example, SDG 4 (Education), SDG 8 (Employment and Decent Work) and especially SDG 5 (Gender equality and empowerment of women and girls [36].

Box 2. SDG 5: promote gender equality and empower all women and girls

- **5.1** End all forms of discrimination against all women and girls everywhere
- **5.2** Eliminate all forms of violence against all women and girls in the public and private spheres, including trafficking and sexual and other types of exploitation
- **5.3** Eliminate all harmful practices, such as child, early and forced marriage and female genital mutilation
- **5.4** Recognize and value unpaid care and domestic work through the provision of public services, infrastructure and social protection policies and the promotion of shared responsibility within the household and the family as nationally appropriate
- **5.5** Ensure women’s full and effective participation and equal opportunities for leadership at all levels of decision-making in political, economic and public life
- **5.6** Ensure universal access to sexual and reproductive health and reproductive rights as agreed in accordance with the Programme of Action of the International Conference on Population and Development and the Beijing Platform for Action and the outcome documents of their review conferences
- **5.a** Undertake reforms to give women equal rights to economic resources… in accordance with national laws
- **5.b** Enhance the use of enabling technology, in particular information and communications technology, to promote the empowerment of women
- **5.c** Adopt and strengthen sound policies and enforceable legislation for the promotion of gender equality and the empowerment of all women and girls at all levels

2. The 2022 *W4H Action Plan* has formally acknowledged the centrality of gender equality in HRH [2, p. 13]. Importantly, the 2022 WHO W4H Action Plan has a monitoring framework with gender-related outputs and indicators whose data sources are the National Health Workforce Account), the SDG database and national reports. Systemic structural gender discrimination and inequality play out in the areas for action described in the W4H Action Plan’s progression model [2, Figure 1, p. iii]. Integrating relevant gender and HRH indicators in all W4H areas for action where relevant would optimize, build and strengthen the workforce Progress towards achieving SDG targets could potentially be integrated in national HRH strategies, action and monitoring and evaluation plans and data bases. This will require political will to achieve SDG 5 targets, since efforts to promote gender equality usually meet with resistance [37]. Operationalizing elements of the 2022 Global Health and Care Worker Compact [which provides technical guidance for Member States and relevant stakeholders on how to protect and safeguard the health, safety, and human rights of health and care workers and ensure that they have safe, supportive enabling work environments [38] can link to SDG 8 targets of decent and productive work, or SDG 5 targets concerning discrimination, violence, the gender segregation of labor and women’s unrecognized unpaid care and domestic work (a structural feature of every economy [39, 40]), and women’s full and effective participation and equal opportunities for leadership.
3. The 2021 *WHO Health Labour Analysis (HLMA) Guidebook* provides guidance on how to analyze the invisible gender dimensions of health labor markets, suggesting gender analysis questions, variables/indicators and data sources. The mapping of *gender orders* and *gender regimes* is a major task of social science research on gender [9] and this implies that gender and workforce analyses should be integrated in HLMA and should map SSGDI in paid and unpaid health work and the flows into, in and out of the health labor market. This would include data on workforce structure and composition (e.g., sex- and age-disaggregated); and describe the *discriminatory* processes, structures and relational patterns and conditions that potentially lead to social closure and that undermine workforce attraction, retention and expansion (e.g., gender descriptive). Most importantly, HLMA should generate evidence describing the real, gendered terms and conditions of health workers’ educational, employment and personal lives to be addressed by gender-transformative HRH policy.

### HRH policy

Gender and HRH analyses can improve policies by making the dynamics, effects and consequences of SSGDI visible as health systems dysfunctions to be addressed by proactive or corrective HRH policy. Revealing masked occupational or professional (social) *closures* for women in health sector is particularly important for understanding workforce flows into, in and around and out of health systems. Relevant contextual gender and HRH evidence should be available for the development of any HRH policy to demonstrate why gender-blind HRH policies would likely be ineffective in countering discrimination and correcting an uneven playing field of gender-segregated labor. Once the operation and consequences of SSGDI are visible, HRH/M policy planners would have the rationale to counter discrimination and inequality though anti-discrimination and substantive equality policies and measures to bring about changes in the real conditions of health workers’ lives and work—for the better. Gender transformative policy will then be workforce transformative. The reader is referred to readings that describe gender-transformative substantive equality and nondiscrimination principles and special measures [22–24, 41].

## What are practical steps to make gender analysis results accessible to and actionable by HRH/M policy planners and managers?

Conducting a gender and HRH analysis is not sufficient to develop and implement effective gender- and workforce-transformative policies. New understandings of gender discrimination, inequality, equal opportunity, nondiscrimination and substantive gender equality will be necessary. To do this, systematic knowledge translation [42] and intersectoral coalition-building are critical. Knowledge translation should feature locally led, iterative, awareness-raising, education, uptake and capacity-building activities in intersectoral coalitions that together can plan evidence-based, gender-transformative strategies and policy. In this way, knowledge translation activities will counter resistance and foster accountability. Such activities require investments of time and money. The findings in Table 1 certainly suggest that investments targeting gender equality and women’s economic empowerment should pay attention to the health workforce, especially since health jobs are potentially important sources of female employment and economic security, economic growth and social development.

## Conclusions

Making the dynamics of SSGDI visible will allow HRH policy planners and managers to craft effective policy and anticipate bottlenecks to workforce entry and flows and transform the actual conditions of health workers’ work and lives for the better. There must be new understandings about systemic structural gender discrimination and inequality in HRH, investment in collecting and using context-specific, sex- and age-disaggregated, gender-descriptive data and translating knowledge into intersectoral policy action to counter their systemic, structural effects and negative consequences for the health workforce. Gender transformative policy based on nondiscrimination and substantive equality principles will be workforce transformative. Global HRH strategy and W4H Action Plan objectives and UHC and SDG goals will more likely be realized.

## Supplementary Information

**Additional file 1.** Systemic structural discrimination and inequality in Senegal’s public health sector.

## Data Availability

Datasets and transcripts from the Sénégal study were destroyed. Data necessary to interpret, replicate and build upon the findings are in unpublished text files and a technical report available from the corresponding author on reasonable request.

## Abbreviations

GDIA: Gender Discrimination and Inequality Analysis
HRH: Human Resources for Health
HRM: Human Resources Management
LMIC: Low- and Middle-Income Countries
NGO: Non-Governmental Organization
SDG: Sustainable Development Goals
SSGDI: Systemic, structural gender discrimination and inequality
UHC: Universal Health Coverage
WHO: World Health Organization
W4H: Working for Health
