# Supplementary material for: Systemic structural gender discrimination and inequality in the health workforce: theoretical lenses for gender analysis, multi-country evidence and implications for implementation and HRH policy
Source: Hum Resour Health. 2023 May 4;21:37. doi: 10.1186/s12960-023-00813-9 (PMC10161468; doi:10.1186/s12960-023-00813-9)
Supplement: Supplementary file 1 — Additional file 1. Systemic structural discrimination and inequality in Senegal’s public health sector. [file 12960_2023_813_MOESM1_ESM.docx]

**Additional data file: Systemic structural discrimination and inequality in Senegal’s public health sector**

**Background:** In 2019, the Senegal Ministry of Health and Social Affairs’ (MOH/SA) collected gender-descriptive and sex- and age-disaggregated data through a Gender Discrimination and Inequality Analysis (GDIA) and a systematic gender and HRH-related policy review, which made aspects of *systemic structural discrimination and inequality* (SSGDI) visible as contributors to female health workers’ absenteeism, deployment-related separation from the spouse/family, stereotyping, sexual and other types of workplace violence, and tension between the reconciliation of family, social and professional responsibilities and labor market employment. SSGDI was evident in the public health sector’s *gender regime* and impeded female health workers’ opportunities for paid work and advancement to higher levels of the public sector bureaucracy. The cross-cultural portability of various concepts was substantiated through GDIA findings: *Gender blindness* and/or unresponsiveness to social embodiment appeared to be a key feature of HRM policy and practice. A *category bias* appeared to exist for female- typed occupations. Exclusion from safe and decent work resulted in occupational and professional *social closure* or exit of female health workers in Senegal’s public health sector.

**1.** **Workforce composition and structure:**

Composition**:** Senegal’s public health personnel are mostly married with children. It is a workforce in its full reproductive years: 90% of female health workers and 73% of male health workers are between 30 and 39 years old (Figure 1). 82% of female and 86% of male health workers are married. 74% of female and 78% of male health workers have children.

**Figure 1: Number and distribution of health workers by age and sex**

**
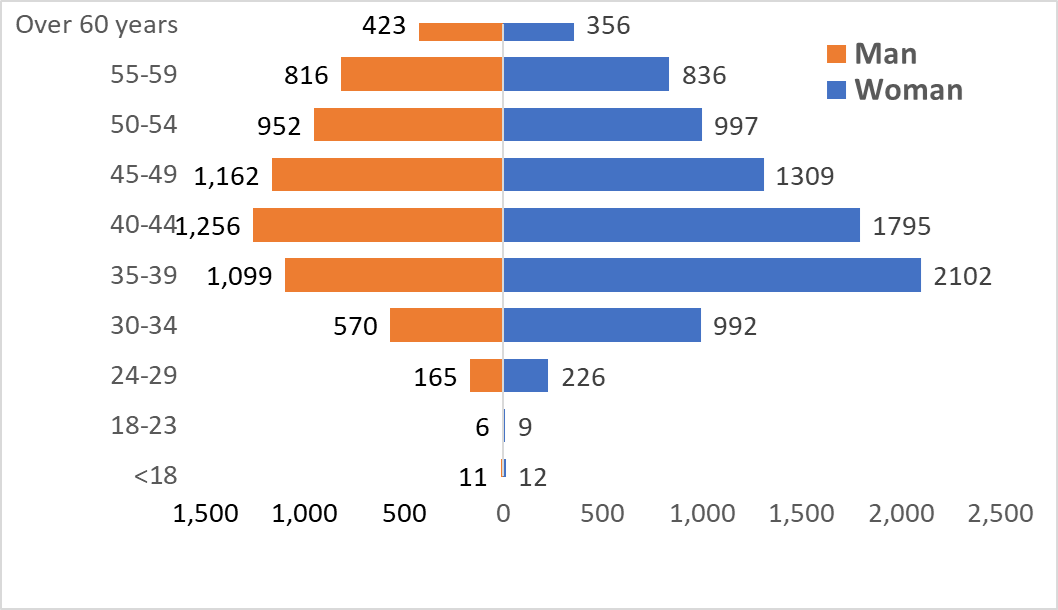
**


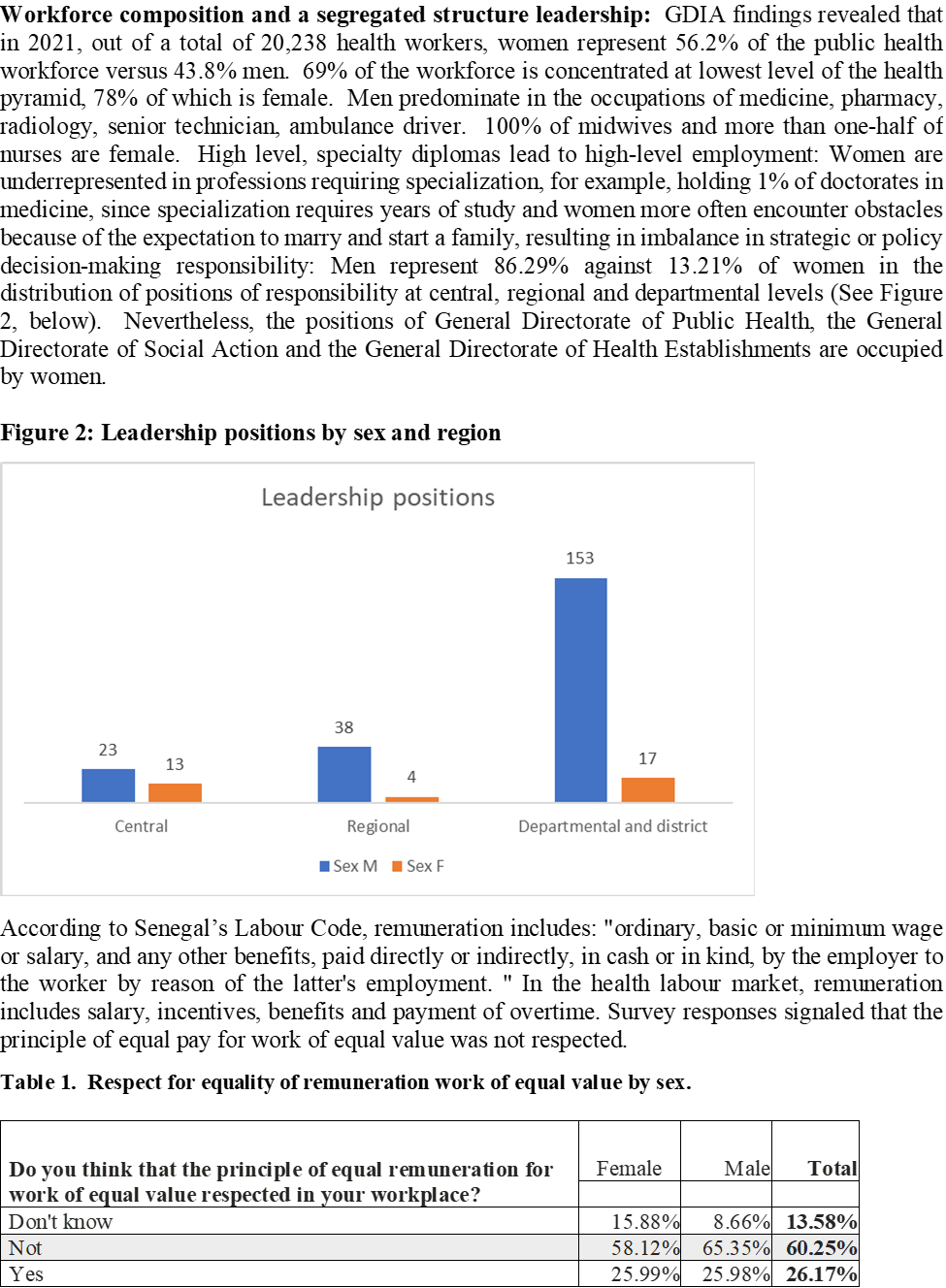


**2. Gender norms, beliefs and ideology** about male primacy and the subordination of women reflected stratification processes. Female health workers’ *private sphere* work (domestic, caring) was highly valued, but health labor market work much less so. There were gendered expectations that good women should follow not lead, submit to aggression and overcome adversity. For example:

Perception that women are illegitimate leaders entered into leadership/management and even treatment decisions: *“The conception is that the woman must follow, not lead a man. When the head is a woman, the community itself will not give her legitimacy…in some health posts where there is a [female] head, men who come for treatment minimize the woman. As a result, a woman chief is very rigorous about what she does, and then men call them hard. Also, the man is not used to receiving orders from the woman, which is the reason why when a woman is their manager, some men do not agree to cooperate.”* (MOH/SA key informant).

“Ideology of domesticity” and workforce participation: Beliefs that hold that a woman is or should be dependent on and protected by a man whose formal duty is to be the breadwinner had a corollary in a belief that a married woman should be limited to the management of the household and children in her primary place, the home. This (traditional) belief is buttressed by Senegal’s Family Code, which requires married women to live with their spouses regardless of where they are deployed for work. This ideology unpinned traditional gender roles and responsibilities and exacerbated conflicts between female health workers’ professional and domestic/ reproductive work: *«The first challenge that the couple must overcome is the maintenance of the family equilibrium. Senegalese society requires the woman to manage the family and the in-laws. In some situations, the woman may manage her husband and children, but also a whole battalion of in-laws, sisters-in-law, brothers-in-law following behind. And what is special about this case is that our society requires more from the wife to manage the in-laws rather than the husband than to manage her in-laws. That is to say, the expectations are much more pronounced on the side of the woman vis-à-vis her in-laws than of the man vis-à-vis his in-laws. So, there is a very heavy burden on the woman. In the majority of cases, the woman will have to be very careful about what she has to do... Imagine a woman who works with a rather tenuous schedule, she leaves her husband to prepare food, for some families it is scandalous!!! Because we're going to say: Look it's like you don't have a wife, you’ve been abandoned, you start doing domestic work..."* (MSAS key informant).

High value placed on women’s domesticity and lower value placed on labor market participation: Men’s labor market contribution was more valued: *"For me, the work of the man is more valued because for society, the man provides more effort than the woman. According to people's understanding, a working woman does nothing in her workplace. We even hear people say to the working woman "you don't do anything, you're only going to work" and yet the woman makes efforts in her workplace, but she is still forced to make efforts at home, while during this time the man is resting”* (Female focus group, Sédhiou).

This situation created frustration for the female health worker who, apart from her professional workload, contributes financially and physically to the expenses of the household. "*You know, I'm in a big family, I work and others work, but when I bring my money, it's like I haven't brought anything, but if men bring their money, it's like it's the whole earth they brought. Sometimes I am even frustrated, but in the conception of the family, the man must work and the woman must stay at home, everything I can do, does not interest them, as long as I am not at home, it is as if I have done nothing. For the family, this absence is more important than the money I give them. People tend to say "she's never there" so that's the presence the family needs. That's how I interpret the situation, only my father understands the situation, but for others it's like they don't see me.”* (Female focus group, Sédhiou).

Gender division of household labor and differential time use conflict: There was a gendered expectation that household expenses were the responsibility of the husband, though few heads of household were able to cover daily expenses alone. A married woman with children often makes significant but unrecognized financial contributions which, because they are not considered a woman’s responsibility, are not recognized: Clothing, uniforms, schoolbooks, medicines, food, electricity, water were covered by both spouses. A comparison of women’s and men’s responsibility for unpaid care and household responsibility found striking differences in responsibilities and time use: 17.6% men versus 81.3% women were responsible for meal preparation; 40% men versus 82.8% women were responsible for house cleaning; 21.5% men versus 76.3% responsible for dishwashing; and 18.9% versus against 74.6% of women were involved in taking care of the spouse.

Work-life conflict: Seventy-three (73%) of female and 69% of male health workers thought that health working hours conflicted with family responsibilities. Some female health workers who are present at the health facility and perform a 24/7 health job and juggle that job with childcare and domestic responsibilities perceived it as unfair that they are still be perceived as absent from their families. [A question for HRM: Absenteeism from what—the health workplace or the family?]

Social closure for female health workers who want a health career: Health worker survey and focus group responses indicated that men are more likely than a woman to make a career in health because women have social/family obligations that prevent them from staying in a job and moving forward: "*There are certain responsibilities charged to the woman, of which we discharge the man, the side taking care of the children, the extended family. Family responsibility is an obstacle to career advancement. We will always have the mentality that it is the man who must work, bring money and make a career."* (Male focus group, Koumpentoum). Expectations of founding a family--marriage, childbearing and child education and care-- were obstacles in a woman's career path in terms of education, employment and professional advancement. *Time poverty* due to the burden of family responsibilities meant deferring positions of leadership/management responsibility: "*So, I admit that it is heavy, it is said that there should be no discrimination between men and women for positions of responsibility, but I think it is more reserved for men. This is a position that requires you to be single or leave your family outright.”* (Female focus group, Kolda. *"Even if you work 12 months out of 12, you cannot claim a career path"* (Female focus group, Vélingara). The responsibilities involved in a female health worker’s reproductive and domestic life changed aspirations. “…*at the end of the doctoral thesis, there were many women who felt obliged to commit themselves to the direction of the family…Women were forced to take a break, get married, have children before considering specialization. Inevitably, our mentalities in Africa have an impact on a woman's career”* (Male focus group, Koumpentoum). There was social pressure *since “when you are married you sometimes want to continue your studies but it is your in-laws that will manage your household and will tell you if you continue your studies or not. So, the woman has to stay at home to manage her household. As soon as you opt for health (a health career), you really have to make sacrifices»"* (Female focus group, Sédhiou).

**NB**: The power of gender ideology and norms on the development and retention of the workforce was evident. Tamale [1] observed that «Domesticity as an ideology is historically and culturally constructed and is closely linked to patriarchy, gender/power relations and the artificial private/public distinction… The way patriarchy defines women is such that their full and wholesome existence depends on getting married, producing children and caring for her family. In Africa, it does not matter whether a woman is a successful politician, possesses three Ph.D.s and runs the most successful business in town; if she has never married and/or is childless, she is perceived to be lacking in a fundamental way. Girl children are raised and socialized into this ideology and few ever question or challenge its basic tenets. Single, childless women carry a permanent stigma like a lodestone about their necks. They are viewed by society as half-baked, even half-human.”

**2. Discrimination based on pregnancy and expected reproductive role**

Glass ceiling/glass escalator: The focus group data suggested that women, especially those who are or are likely to become pregnant, were disadvantaged in terms of recruitment, hiring and promotion. Anticipating that female health workers will take maternity leave, and be responsible for children’s and family care with its attendant work absences when they get back from leave, hiring managers favored male candidates especially for management/decision-making positions: *"Men are prioritized over women because women become pregnant, they breastfeed, they have problems with the sick child etc. So, you choose men more than women because it's a question we experienced at the hospital of Touba …When the hospital had just started, the director of the hospital said he would recruit more male nurses than females.* ». *«"It's a problem that's there. In some regions, they will prefer to appoint a nursing assistant rather than a midwife, to head of post."* (MOH/SA female focus group).

Facility managers were perceived to react negatively when a health worker became pregnant because it constitutes a blockage in the continuity of care. *"When people know that there is a lady who is pregnant, we think about her maternity leave because she will be absent for at least four months and she will not be replaced. Now it is the head of department who has to plug the absence”* (Male focus group, Vélingara). Because of the emphasis of meeting targets, pregnant employees fear disapproval: "*As she just said, here, being pregnant is a constraint. While the person when is pregnant, must be jovial, happy, but it is the opposite, you are afraid of the eye whose superior will look at you. Right now, at the hospital level, there is tension, because there are agents who are pregnant… I went to see the doctor to put an order for a medical rest, but what he told me, myself as a woman, I was flabbergasted, because he told me "But in the department, all women are pregnant, it cannot be done,*" *so, they, they think only of the service, it is as if no one should get pregnant, that women have no life outside the service”* (Focus group, women, Sédhiou). As a key informant observed, breaks from education or work result in it taking 10 or 12 years for a female health worker to earn a generalist degree to a male’s 7 years. The discriminatory nature of this situation was signaled by both male and female health workers who attested that men did not face this health career disadvantage.

Abridged maternity leave and informal accommodation to breastfeeding and childcare: Pregnant health workers sometimes found it difficult to take full maternity leave because of the lack of staff, regardless of Senegal’s legal maternity protection. Often facility staff informally and cooperatively organized themselves to cover for or lighten the workload for pregnant or lactating health workers, such as for night duty. Issues like these were usually handled on a case-by-case basis between worker and manager who would find try to find individual solutions to an organizational problem.

Work spaces: There was no space reserved for breastfeeding or childcare at work. Breastfeeding health workers made personal accommodations, such as asking their domestic worker to bring the child to the health facility to take a 15-minute break, or going home during break hours to breastfeed the child. Health workers believed that the fact that they remain in their workplace without breastfeeding their children was a factor that undermined the credibility of their recommendation to clients regarding exclusive breastfeeding.

**4. Insecure, unsafe and “indecent” working conditions:** Because of COVID 19 in 2020, there was lower attendance at health facilities because of the reluctance of populations to visit facilities perceived to be the place of virus transmission. However, lower attendance did not lessen an additional workload because *health workers* had to travel into communities to raise awareness about infection prevention measures and to care for suspected cases. *Health workers* faced constant risk and fear of being infected with the virus. Focus group respondents also mentioned that health facilities allow free access to the public, which exposes them to various forms of workplace violence. Typically, older/elderly people are hired to guard the doors to keep out “*smokers of Indian hemp, the alcoholics, drunkards…Sometimes we lock ourselves up to prevent us from being attacked.”* (Female focus group, Kolda). While this presented a problem of lack of personal safety and security, insecurity extended to housing. Indeed, insecure housing in remote areas can push women to leave the health sector: "*There's the problem of insecurity, I don't know if that answers the question. Working in areas where there is no electricity, they do not have decent housing. There is a woman who told me last time that her house does not have a lock”* (Female focus group, Kolda). Nurses and midwives also traveled on motorcycles on often unpassable roads for community outreach and the jarring bumps from potholes are believed to cause miscarriages: *“It is not only the marriage that gives tranquility, there are pregnancies. Imagine a woman who has the desire to give birth and ends up in with repeated miscarriages finally, she will say to herself, is the work worth it?* (Female focus group, Sédhiou).

**5. Workplace abuse and sexual violence:** Violence against women in, around and traveling to work was mediated by implicit and enacted beliefs that violence against women was legitimate but should not be talked about. Violence and harassment by patients, relatives and other health workers were common: *“Gender-based violence is often verbal or physical violence. But it must be recognized that health workers, especially women, are victims of this violence. Indeed, women health workers are often verbally harassed by those accompanying patients during childbirth and consultations, especially on holidays. There is violence that exists.”* (Female focus group, Tamba). *“Some have used verbal abuse when you take time to consult. It's gender harassment. As part of free medication for children under five, if you don't have the medication, some parents verbally assault you why you don't have the medication”* (Female focus group, Vélingara). *"It's physical violence, because more and more people tend to beat us because we're women. And most of the time, they are accompanying the husband or brother and who are often authoritarian. And sometimes there is also verbal violence with stereotypes about health workers. No one says how often we are insulted with all the names… you see that it is verbal harassment or verbal aggression that we suffer every day and everywhere in Senegal.»* (Key informant, Dakar). *"As soon as they see that you're short, they say you don't know how to do anything, or that you're just a child.”* (Female focus group, Kolda)…” *“It is common to see altercations; insults; physical violence; verbal abuse. There is a lot of it” (*Key informant, Union president).

Sexual violence/sex-based harassment: The term sex-based harassment [2] includes three categories of behavior: *Gender harassment* (Behavior that degrades and denies the targets’ professional standing based on gender rather than solicits sexual cooperation); *unwanted sexual attention* (Unwelcome and un-reciprocal sexual behaviors aimed at establishing some form of sexual relationship); and *sexual coercion* (Unwelcome sexual advances coupled with bribery, forced consent or assault, intimidation, or threats in order to establish a sexual relationship). Sexual harassment was perceived as a problem mainly affecting female health workers, though unwanted sexual attention by female patients or staff towards male health workers was mentioned. Unwanted sexual attention and coercion by supervisors/ managers was such that 38% of female and 31% of male health workers indicated “yes” to a question on whether there should be a MOH/SA policy that prohibits a sexual or romantic relationship between a supervisor/manager and junior staff. Fearing retaliation appears reasonable: *“Sexual coercion has consequences on the person who refuses to cooperate…the consequences on the person are serious. For example, it can be psychological…others even go so far as to leave their posts. It's even going on here--we’ve lived it. Often it is the authorities who are the perpetrators of these injustices. These acts are incomprehensible: We have studied like them and we have also graduated like them, so it shouldn’t happen this way. It is not because one is the boss that one abuses power»* (MOH/SA female focus group). Harassing behaviors created a hostile work environment and abridge female health workers’ equality of opportunity, work productivity, security, labor market participation and career advancement, especially trainees and health workers with short-term contracts.

Silence is the norm: The MOHSA Human Resources (HR) does not have formal sexual harassment prevention and response mechanisms. Health workers usually did not report because of fear of disbelief, stigma and professional and personal repercussions. The evidence suggested that the female target was held responsible for having caused the harassment (i.e., the victim is blamed) and that the harasser has impunity, as “*there are societal norms that give man this power. Sometimes he even interprets religion for their benefit by stipulating that he has the right of life or death over you. The main problem is the lack of consideration from superiors. A woman told me ‘I am not safe at any noise I jump. Even if I see a man approaching me, I'm afraid.’ So, on the professional side, there is precariousness and the environment, because many midwives who are not recruited by the public service and are contractor with contracts that must be renewed every year. Now, the person who has to renew it can harass you and if you denounce it, there will be a national coalition against you because you have touched a higher professional category and the victims are often afraid that he will retaliate on their professional career. So that's the dilemma. »* (Key informant, association president). Yet, *"to say that someone assaulted or raped me is frowned on by society. For example, if you are single, you will have a hard time finding a husband because the story will resurface and they will then ask questions about virginity. So, suddenly people are afraid of these consequences, even with 100 rapes they will say nothing. And it's the same case in our homes with the uncles who rape their nieces and no one talks about it. It is the perpetrator who should be condemned, but society condemns the victim. »* (MOH/SA female focus group). *“I know that sexual harassment is very frequent and often it results in intimidation, assault or a threat to be transferred to the interior in areas where really it is very difficult, to be sidelined during training, promotions”* (Key informant, association president).

Sexual harassment, occupational (social) closure and workforce exit: "*Above all it is the stigmatization. Even your grandchildren and great grandchildren will know the story. Most often the victim of harassment is a victim of blame, we do not even believe you and there are psychological consequences and in order not to be labeled, you prefer to be silent and not to denounce, keep it internally and suffer"* (Female focus group, Tamba) *“The environment can also push [you] to leave, for example if the manager does not give you peace, you will not stay there. A person stays somewhere because of the interests in that sector”* (Female focus group, Sédhiou).

**6. Occupational segregation and category bias:** In Senegal’s public health sector, “occupational ghettos” comprised of female-typed health occupations (e.g., nurse or midwife) and male-typed jobs (physician) which featured differential professional work terms and job conditions. For example, the female-typed paramedical cadre of nurses and midwives had to use motorcycle transportation on bumpy roads to conduct rural health campaigns, and lived in insecure housing, exposing them to physical danger. Those in female-typed occupations performed work at night (night duty) often as unpaid overtime, which did not lead to promotion. Night duty involved chronic nightly disruptions of family life (the private sphere) because most female health workers in remote rural areas had no one to take care of their children. Nurses, midwives and matrons sometimes brought their young children to work as a way to reconcile these working hours with family life. Female health workers were also exposed to violence and harassment during the day and night.

In contrast, medical doctors (mostly male) did not typically perform these undesirable and inconvenient tasks or experience these conditions. Occupations at the upper echelons of the health system hierarchy, where male staff were concentrated, enjoyed “class” benefits, such as better/secure housing; access to credentialing scholarships; and faster integration in the more secure public service. In this way, gender and occupational class intersected in a “category bias” or a gendered class bias favoring male health workers. A category bias [3] is evident "when an entire group of workers, which happens to be predominantly female, is treated less favorably than another group, which happens to be predominantly male. This allows an entire group to be denied certain rights and protections because of the sex-based structure of professions.” According to a president of a professional association, “*At the Ministry of Health, everything is in the hands of doctors, everything revolves around doctors.”*  The paramedical occupations (midwife, nurse) conferred “second class citizenship” which made health labor market less attractive for them.

**7. Formal institutional unresponsiveness to social embodiment, discrimination and bias**: At the time of the GDIA, MOHSA HRM policies were blind to gender ideology, expectation, roles and responsibilities, but in the process of overhauling its HR policies and guidelines in light of GDIA findings. The MOHSA *Gender Mainstreaming Plan* noted that spousal and family separation is a bigger problem for female than male health workers. Gender-blindness to *social embodiment*, for example, to the effects and consequences of its deployment policy, gave rise to work conditions that a MOHSA director once described as “inhumane.” Family and spousal separation and work-life conflict became extremely destabilizing dynamics which contributed to guilt, worry and absenteeism. Work was fraught with tensions whereby the female health worker was perceived by managers/co-workers as absent from the job, while professional responsibilities caused the female health worker to be perceived by spouse and extended families to be absent from their home. Female health workers engaged in stressful “gymnastics” to juggle two sets of conflicting expectations and commitments. Health workers have sought institutional support, but *"if you go to the Ministry level to tell them about your marital status, telling them that 'my husband is asking me for a divorce because of the situation, they start laughing, taking her out of their offices the only thing we want to do is cry. You see women on the stairs crying…Imagine a woman whose husband threatens to repudiate [her], she comes to the ministry level to have a solution and they start laughing, that's what happens." Me, the last time I went to the ministry, I could not even say what brought me, I was consoling a lady who came from Kidira. Her husband had given her a month to join the family or divorce.”* (Female focus group, Sédhiou).

**8. Social closure and labor market exit:** The need for gender-aware and -responsive institutional HR policies, reflecting nondiscrimination and substantive equality, was made evident through the GDIA data. In a gendered organization with HRM policies that are blind to social embodiment, bias and discrimination, the *inequality-generating* processes involved in gender differentiation, stratification and exclusion contributed to *occupational and professional (social) closure* at different sites in health education and employment systems. Opportunities for a health career are closed off at various career points, not to mention organizational/sectoral leadership. The high value placed on female *domesticity* and non-recognition and undervaluation of health labor market participation and earnings, along with the pressures, conflicts and personal and social costs experienced under present work conditions, were simply too high for many female health workers. These factors made the public health labor market unattractive and contributed to unsustainable work-life conflicts and social destabilizations that ended in exit from the health labor market.

**References**

1. **Ideology of Domesticity:** Tamale S. Gender Trauma in Africa: Enhancing Women’s Links to Resources Cairo, Council for the Development of Social Science Research in Africa (CODESRIA) and the Arab Research Centre (ARC); 2002, Presentation for African Gender Research in the New Millennium: Perspectives, Directions and Challenges. <https://www.jstor.org/stable/27607908>
2. **Sex-based harassment**: Fitzgerald, L. F., Gelfand, M. J., & Drasgow, F. (1995). Measuring sexual harassment: Theoretical and psychometric advances. Basic and Applied Social Psychology, 17(4), 425–445. https://doi.org/10.1207/s15324834basp1704_2; and International Center of Research on Women. costs of Sex-Based Harassment on Businesses: An In-Depth Look at the Workplace. 2010. ICRW_SBHDonorBrief_v4_WebReady.pdf
3. **Category bias**: Standing H. Gender—a missing dimension in human resource policy and planning for health reforms. Human Res Health Development Journal. 2000;4:27–42. (PDF) Gender – a Missing Dimension in Human Resource Policy and Planning for Health Reforms (researchgate.net)
4. **Social Closure:** Catherine Albiston C and Green TK. Social Closure Discrimination, 2018. 39 Berkeley J. Emp. & Lab. L. p.5. Social Closure Discrimination by Catherine Albiston, Tristin K. Green: SSRN.
